# Anti-obesity effects of olivetol in adult zebrafish model induced by short-term high-fat diet

**DOI:** 10.1038/s41598-023-44462-3

**Published:** 2023-10-27

**Authors:** Andukuri Reshma, T. Tamilanban, V. Chitra, Vetriselvan Subramaniyan, Gaurav Gupta, Neeraj Kumar Fuloria, Mahendran Sekar, Shivkanya Fuloria, Rakesh Sahu, J. Narayanan, Srikumar Chakravarthy, Siddharthan Selvaraj

**Affiliations:** 1 Department of Pharmacology, SRM College of Pharmacy, SRM Institute of Science and Technology, Kattankulathur, Tamil Nadu 603203 India; 2 Pharmacology Unit, Jeffrey Cheah School of Medicine and Health Sciences, Monash University, Jalan Lagoon Selatan, 47500 Bandar Sunway, Selangor Darul Ehsan Malaysia; 3 Centre for Global Health Research, Saveetha Medical College and Hospital, Saveetha University, Chennai, 602105, India; 4 School of Pharmacy, Graphic Era Hill University, Dehradun, 248007 India; 5 School of Pharmacy, Suresh Gyan Vihar University, Jagatpura, Jaipur 302017 India; 6 Faculty of Pharmacy, AIMST University, 08100 Bedong, Kedah Malaysia; 7 School of Pharmacy, Monash University Malaysia , Jalan Lagoon Selatan, 47500 Bandar Sunway, Selangor Darul Ehsan Malaysia; 8 CSIR-NEIST, Jorhat, Assam 785006 India; 9 SEGi University, Jalan Teknologi, Taman Sains Selangor, Kota Damansara, PJU 5, 47810 Petaling Jaya, Selangor Malaysia; 10 Faculty of Dentistry, AIMST University, 08100 Bedong, Kedah Malaysia

**Keywords:** Preclinical research, Nutrition

## Abstract

Obesity is a complex disease caused by various factors, and synthetic drugs used to treat it can have side effects. Natural compounds, such as olivetol, could be a promising alternative. Olivetol is a substance found in certain lichen species and has anti-inflammatory and anti-cancer properties. In this study, researchers conducted in-silico molecular docking studies and found that olivetol had significant binding affinity with receptors involved in obesity. They also investigated the effects of olivetol on a diet-induced obese zebrafish model and found that high doses of olivetol reduced excessive fat accumulation and triglyceride and lipid accumulation. The low dose of olivetol showed a significant reduction in liver enzymes' levels. However, the high dose of olivetol resulted in a significant increase in HMG-CoA levels. These results suggest that olivetol may be a promising anti-obesity agent for the treatment of hyperlipidemia-related disorders, but further research is necessary to understand its full effects on the body.

## Introduction

**Figure 1:**
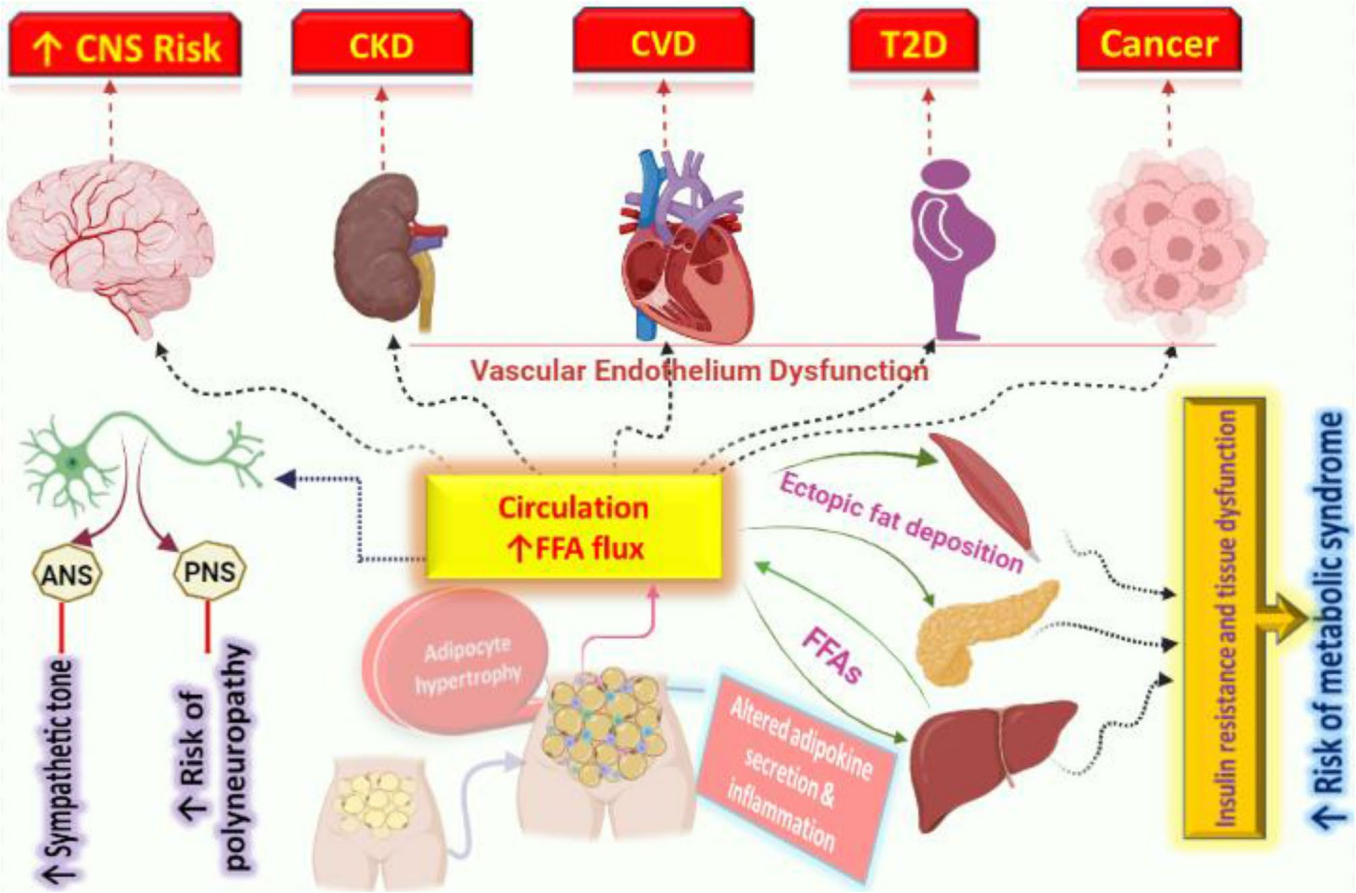
Obesity is linked to several diseases including cardiovascular disease, type 2 diabetes, and non-alcoholic fatty liver disease. High levels of LDLc, or "bad" cholesterol, and increased FFA flux are two mechanisms linking obesity to these diseases. LDLc can contribute to the formation of plaque in blood vessels, while excess FFA can lead to insulin resistance and inflammation. Lifestyle changes such as diet and exercise can help manage obesity and reduce the risk of associated diseases.

Obesity is becoming more common due to global pollution as the number of chemical compounds in the environment continues to increase^1^. The interplay of genetic, metabolic, social, behavioral, and cultural factors leads to the development of obesity, which is a complex, multifactorial disease^2^. The most common cause of obesity is an imbalance between energy intake (dietary intake) and energy expenditure (energy loss via metabolic and physical activity). The etiology of obesity is extremely complex and includes genetic, physiological, environmental, psychological, social, economic, and even political factors that interact to varying degrees to promote its development^3^. It is widely recognized that obesity presents a serious threat to public health, as it increases the risks of a variety of diseases such as type II diabetes, cardiovascular disease, hyperlipidemia, hypertension, stroke, colon cancer, and degenerative arthritis^4,5^. Treatment regimens for obesity include exercise, dietary changes, and anti-obesity medications. Decreased HDL-c, increased LDL-c, and triglyceride levels are thought to be indicators of obesity (Fig. 1). In most instances, there is no relationship between having high cholesterol levels and being obese. Pregnancy, hypothyroidism, polycystic ovarian syndrome, and certain medications raise LDL cholesterol levels but lower HDL cholesterol is among the conditions that cause elevated cholesterol levels^6^.

**Figure 2:**
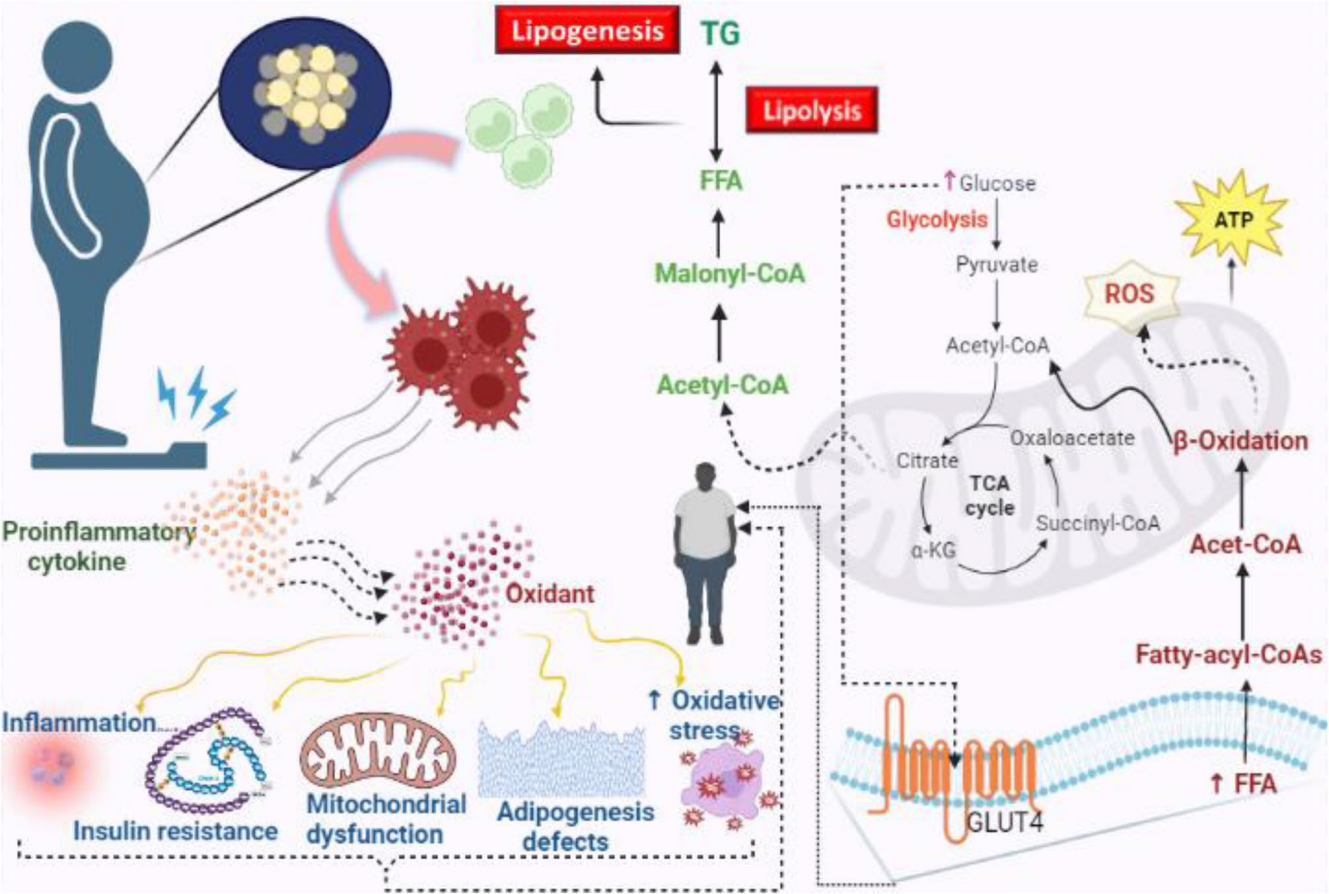
Alterations in metabolism associated with obesity are linked to increased activity of the HMG-CoA enzyme and oxidative stress. HMG-CoA is a key enzyme involved in cholesterol synthesis, and its increased activity in obesity can lead to elevated levels of LDLc.

Obesity has a complicated etiology that involves complex interactions among genetics, hormones, and the environment^7^. The pathophysiology of obesity has been linked to numerous candidate genes; however, the results are inconsistent. These genes include the beta-3-adrenergic receptor gene, peroxisome-proliferator-activated receptor gamma 2 gene, chromosome 10p, melanocortin-4 receptor gene, and other genetic polymorphisms^8^. The most efficient treatment for obesity is hydroxy-methyl glutaryl co-enzyme reductase (HMG CoA) inhibitors, which have been found to reduce both body and liver fat that obese rats have accumulated (Fig. 2). HMG CoA reductase inhibitors are commonly used in the treatment plans for obesity and have been shown to be effective^9^.

In-depth studies have investigated the roles of genes associated with obesity and the responses of rodents to high-calorie and high-fat diets. These studies have typically shown that food regimens, genetic makeup, and knockout gene functioning can all influence the obesity phenotype. This suggests that developing and investigating models of obesity that are influenced by genetics and nutrition is essential^10^. It is clear from this that models for diet- and genetics-induced obesity are crucial to design and evaluate. Rodent model trials are generally labor-intensive, require infrastructure support, and are very expensive, although they have significantly contributed to our understanding of human obesity^11^.

There has been significant research attention focused on exploring the therapeutic potential of cannabinoids, specifically in relation to pain management, Anti-inflammatory action, and the maintenance of metabolic Haemostasis^12^. Olivetol, classified as a resorcinolic phenol, acts as a foundational compound for the synthesis of cannabinoids. Commonly identified as 5-pentylresorcinol, it is naturally present in lichen species. Within the extensive array of natural plant compounds, polyphenols and phenols assume a prominent role due to their significant contributions as agents with anti-carcinogenic, anti-inflammatory, antioxidant, and antiviral properties. Reactive oxygen species (ROS) and the maintenance of metabolic balance are contributing factors to the dysfunction of adipose tissue^13^.

Herbal compounds are considered a crucial focus for drug development due to the diverse range of phytoconstituents they contain, along with their limited adverse effects^14^. Medicinal Plants are the sources of many bioactive compounds which shows results in managing obesity. Within the secondary metabolites of plants, particularly polyphenols and terpenoids, there is notable evidence of their efficacy in promoting effective weight management^15–17^. Certain alkaloids exhibit promising potential for treating obesity; however, the considerable toxicity associated with the majority of these compounds limits their applicability. Phytoconstituents exert diverse anti-obesity effects, functioning through various mechanisms. These include the inhibition of enzymes responsible for metabolizing lipids and carbohydrates, the suppression of appetite and adipogenesis, hindrance of lipid absorption, and augmentation of energy metabolism^18^. The involvement of oxidative stress in the development of obesity and its related risk factors were reported in many in-vivo, epidemiological and clinical studies^19^.

Oxidative stress has the potential to initiate obesity by promoting the accumulation of white adipose tissue (WAT) and influencing food consumption^19^. This stressor can prompt heightened proliferation of preadipocytes, facilitate adipocyte differentiation, and contribute to the enlargement of mature adipocyte^20–23^. Reactive oxygen species (ROS) have been identified as contributors to the regulation of body weight through their distinct impacts on hypothalamic neurons responsible for governing sensations of satiety and hunger behaviours^24^. Obesity itself can initiate widespread oxidative stress within the body through a variety of biochemical mechanisms. These mechanisms include the generation of superoxide from NADPH oxidases (NOX), oxidative phosphorylation, auto-oxidation of glyceraldehyde, activation of protein kinase C (PKC), and engagement of the polyol and hexosamine pathways^25^. Additional factors that contribute to oxidative stress in the context of obesity encompass hyperleptinemia, impaired tissue function, diminished antioxidant defences, persistent inflammation, and the generation of reactive oxygen species (ROS) following meals^26,27^.

In the pursuit of developing novel pharmaceuticals for combating obesity, the endogenous cannabinoid system (ECS) continues to be a prominent area of interest. The endogenous cannabinoid system (ECS) is extensively present in both the central nervous system and peripheral tissues. Its involvement in physiological processes related to food consumption and energy balance primarily occurs through the cannabinoid type 1 receptor (CB1). Inhibition of the CB1 receptor leads to weight reduction, enhances cardio metabolic risk factors and insulin sensitivity, and produces positive metabolic outcomes. This increases the possibility of utilizing CB1 receptor blockade as a therapeutic approach for addressing obesity.

Olivetol, functioning as a natural phytomolecule, exhibits antioxidant attributes that can effectively diminish reactive oxygen species (ROS)^28^. Furthermore, olivetol acts as a competitive inhibitor of cannabinoid receptors CB1 and CB2. Hence, the primary focus of this research is to assess the anti-obesity effects of olivetol, examining its capability to inhibit cannabinoid receptors while concurrently exploring its potential to diminish reactive oxygen species (ROS).

To complement the current rodent models, it has been proposed to create simple and economical animal models of obesity. Recent studies on worms, flies, and zebrafish energy homeostasis have revealed that these less complex creatures can be used to explore the metabolic pathways underlying obesity^29,30^. Zebrafish are vertebrates, and unlike worms and flies, they share several anatomical characteristics with humans. For instance, the skeletal muscle, adipose tissue, and digestive organs of zebrafish are physically comparable to those of humans^30–33^. A zebrafish model for diet-induced obesity was developed by Takehiko Okada et al., and the model was validated using a variety of techniques, including diet therapies, histological, and biochemical examinations. This research showed common pathophysiological pathways between diet-induced obesity in zebrafish and obese mammals, indicating that zebrafish may be a more useful model for diet-induced obesity than previously thought^10^. In particular lichen species, Olivetol is a naturally occurring organic compound that can easily be extracted.

To better understand human obesity and disorders associated with it such as type 2 diabetes, visceral adiposity, and atherosclerosis, zebrafish (Danio rerio) are being employed as models. Oka et al. established the DIO model in zebrafish in 2010. To induce obesity in adult Zebrafish, 60 mg of Artemia/brine shrimp (150 cal) were fed to each fish daily, and for non-induced fish, 20 cal of the same feed in 5 mg form were used. In comparison with zebrafish that were fed normally, the overfed fish had higher BMIs, hypertriglyceridemia, and hepatic steatosis. This model had been validated by several biochemical and histological techniques, which also showed that obese mammals and DIO zebrafish share most of the physiological mechanisms associated with obesity. In addition to overfeeding shrimp, various methods have been employed to make Zebrafish obese. Meguro et al. developed 20% corn oil or lard-based high-fat Zebrafish diets in 2015. They demonstrated that these high-fat diets cause obesity in zebrafish^34^.

According to research, obesity brought on by the consumption of a high-fat diet differs significantly from that caused by consuming an excessive amount of a normal-fat diet. A 2017 study by Landgraf et al. compared the metabolic profile of zebrafish overfed with a high-fat diet (HFD; 59% fat) and zebrafish overfed with a normal fat diet (NFD; shrimp, 22% fat) to generate obese zebrafish. Although both diets led to adiposity, fish overfed with NFD were metabolically healthier than fish overfed with HFD, which were shown to have obesity-related indicators such as elevated glucose intolerance, fatty liver, and a preferential increase in visceral fat^35^.

BMI, triglyceride, cholesterol, and HMG-CoA reductase levels are common measurements used to confirm obesity^10^. Using oil red O staining, lipid and triglyceride accumulations were observed across the entire body of zebrafish^36–38^. The DIO model has been previously used to investigate the anti-obesity effects of natural substances. In a 2015 study by Meguro et al., green tea extract was given to DIO zebrafish. The extract was revealed to have an anti-obesity effect, and this research also showed that a fat-rich diet with low amounts of protein and carbohydrates induced excessive body fat accumulation in zebrafish in ways that were similar to those that occur in mammals^39^. Using DIO zebrafish and mouse models, different research on the anti-obesity properties of Palmariamollis (PM) revealed that it could drastically lower visceral adiposity and hepatic steatosis^10^. Thus, it has been proven that the DIO model of zebrafish is suitable for studying the impacts of several supplements on the accumulation of body fat^40^. Adult zebrafish were provided with a diet high in fat and calories to induce the DIO model for this investigation. Using the well-known DIO model, Olivetol’s anti-obesity properties were evaluated, and the outcomes were compared with those of a conventional statin medicine. The current study would be a starting point for further investigation to find a potential phytochemical that may be used to treat obesity.

The objective of the current research was to investigate the hypothesis that obesity increases hepatic cholesterol production. The activity of the rate-limiting enzymatic step in cholesterol biosynthesis, 3-hydroxy-3-methylglutaryl coenzyme A (HMG-CoA) reductase, was assayed in liver microsomes from morbidly obese zebrafish and normal weight controls^41^.

The prevailing strategy for the management of hypercholesterolemia is the use of HMG-CoA reductase inhibitors which work by inhibiting cholesterol synthesis by HMG-CoA reductase in the liver and removal of excess cholesterol level in peripheral circulation by several mechanisms of reverse cholesterol transport^41^.

CCK or Cholecystokinin plays a role in the acute regulation of appetite and energy intake and abnormalities in CCK, or its actions, on gastrointestinal function and appetite, may contribute to the development and maintenance of obesity^42^.

GLP-1, a 30- or 31-amino-acid-long peptide hormone, is a gastrointestinal peptide secreted by the intestinal tract that potentiates insulin release and reduces glucagon’s concentration in physiological conditions. GLP-1 is necessary for standard glucose tolerance and functions through specific GLP-1Rs, which belong to the G-protein-coupled glucagon receptor family, expressed in islet β-cells and stomach, small intestine, mucosa, heart, and other cell types^43,44^.

Myeloperoxidase (MPO) is abundantly expressed in neutrophils and has been implicated in the initiation of the inflammatory response in adipose tissue. Therefore the following four receptors were selected for the study which includes HMG Co-A, CCK, GLP-1, and MPO.

## Materials and methods

The test compound evaluated was Olivetol. Molecular docking scores were compared for receptors such as HMG Co-A, CCK, GLP-1, and MPO. The docking scores were used to determine the compound's ADMET properties and evaluated using the in-vivo zebrafish model^23^.

### In-silico screening

#### Docking

The target protein selected for analysis was downloaded in ".pdb" format from the RCSB PDB (Protein Data Bank). The ligand structure was designed using the ChemSketch molecular modeling program. The interaction between proteins such as CCK, HMG-CoA, GLP, and MPO with the PDB ID: 1D6G, 1HW9, 5VAI, and 5FIW was examined. The ligand was screened and saved in "mol" format. To run the AutoDock 1.5.7 software, pdbqt files of the ligand and protein were required^24^. The results were then analyzed, and the interactions were observed using the Molegro molecular viewer^45^.

#### Preparation of protein

The Protein Data Bank (PDB) was used to search and download the 2D structure of the protein in pdb format. The file was imported, and all residues were removed using the hierarchy option. Polar hydrogens were added, and the file was saved in .pdb format. The optimized target was then opened in the PyRx tool and converted to “.pdbqt” format^46^.

#### Preparation of ligand

The PubChem ligand database was used to search for ligands and identify their 2D structure. The file was saved in mol format. Next, the saved file was imported into Molegro molecular viewer and the molecule was exported in .pdb format^47^.

#### Virtual screening of docking analysis

The selected ligand with optimized structures was analyzed and screened by running Autodock software on the target proteins (PDB ID: 1HW9, 1D6G, 5VAI, and 5FIW). After docking, the binding affinity results were obtained. The target protein was retrieved from RCSB PDB (Protein Data Bank) in .pdb format^48^.

### Pharmacokinetic analysis

#### Calculation of molecular properties and bioactivity scores

Molinspiration Cheminformatics was used to manipulate and process the ligand molecule, including converting SMILES, normalizing molecules, and calculating various molecular properties. The canonical SMILES of the ligand were entered to calculate the molecular properties and bioactivity score using Molinspiration cheminformatics software^49^.

#### Swiss ADME

The assessment of ADME was generated using the SWISS ADME web tool. The one-panel-per-molecule is headed by the name of the molecule and divided into different sections. The chemical structure of Olivetol is initially described, followed by its canonical SMILES and bioavailability. The boiled-egg representation is an intuitive method to simultaneously predict two key parameters, namely, passive gastrointestinal absorption (HIA) and brain access (BBB). The egg-shaped classification plot includes the yolk, describing the physiochemical space for highly probable BBB permeation, and the white portion describes the physicochemical space for highly probable human gastrointestinal absorption (HIA)^50^.

#### In-vivo study

The test drug Olivetol was purchased from SISCO, South India Surgical Co. Ltd. The medication was dissolved in water and given at appropriate doses. Zebrafish were chosen as the animal model to investigate Olivetol’s anti-obesity effects since it is a suitable alternative animal model for such research. The zebrafish were procured from Local supplier. The corn oil was obtained from Sigma Aldrich, India. The feeding and artemia for the fishes were standard diet feed obtained from the supplier. 

### Ethics statement

All procedures complied with the regulations covering animal experimentation within SRM Institute of Science and Technology (SRMIST) Institutional Animal Ethics Committee (IAEC), India. The reference number for this compliance is IAEC/302/2022. The authors have confirmed that this study was reported in accordance with ARRIVE guidelines.

### Establishment of adult zebrafish model of diet-induced obesity

Method: Ninety fish were fed brine shrimp for seventy days, along with a regular diet and 20% corn oil feed, providing 450 cal per fish per day. It was calculated that 1 mg of shrimp is equal to 5 cal (450 cal equal 90 mg of shrimp). The standard meal was delivered twice daily, and in addition to 20% corn oil at the end of the day, the zebrafish also received 30 mg of artemia three times per day. After feeding, any leftover food was removed, and the water was changed after fifteen minutes. The zebrafish were then separated into groups and placed in separate tanks^11^. Table 1 shows how the different groups were created in the in-vivo study.

**Table 1:** Grouping for in-vivo studies.

Group No	Group	Category of Fish	Drug treatment	No.of. animals	No.of. days
I	Normal group	Non-obese	–	12	14
II	Negative control (Obese)	Obesity-induced fish	–	12	14
III	Positive control (Standard)	Obesity-induced fish	Simvastatin (0.1 mg/1 ml)	12	14
IV	Low dose of Olivetol	Obesity-induced fish	Low dose of Olivetol (0.001 g in 1 ml)	12	14
V	High dose of Olivetol	Obesity-induced fish	High dose of Olivetol (0.001 g in 0.5 ml)	12	14

### Grouping for in-vivo studies

#### Treatment with Olivetol

Different groups of obese Zebrafish were divided and given Olivetol treatment at two different concentrations: 0.001 g dissolved in 1 ml distilled water as the low dose, and 0.001 g drug dissolved in 0.5 ml distilled water as the high dose concentration. For 14 days, the fish were exposed to Olivetol for four hours each day. After the treatment, the water in the tank was changed, and all the fish were given normal feed at regular intervals. The OECD guidelines for using adult zebrafish were followed to determine the dose^51^.

#### Treatment with standard drug Simvastatin

Simvastatin was bought from a neighborhood pharmacy, and 1 mg of it was weighed in 1 ml of distilled water to make the stock solution. Simvastatin was administered to the fish in the positive control group at a dose of 1mg/ml (stock concentration) per liter of water. For 14 days, the medication was administered to the zebrafish for four hours each day. After treatment, the water in the tank was changed, and the fish were fed at regular intervals^52^.

#### Biochemical analysis

The methods for the estimation of cholesterol, triglyceride, HMG-CoA reductase assay, AST, ALT, and ALP were the same as those mentioned by Nagaraj, Anushree, et al. in 2021^51^. The kits for the assay of AST, ALT and ALP were procured from Sigma Aldrich, India^52^.

#### Oil Red O staining

With 4% formaldehyde solution in PBS, the fish were preserved. According to Oka T et al. in 2010, the fixed samples were quickly frozen in liquid nitrogen-cooled isopentane and the following procedures were carried out^11^. The histopathological analysis was carried out under 40 × magnification.

### Statistical analysis

Data were analyzed by GraphPad Prism (GraphPad Prism V7.0). One-way ANOVA followed by NewmaneKeul's post hoc test for multiple comparisons was utilised. The p values less than 0.05 were considered significant. Results are expressed as mean ± standard deviation (SD).

## Results

### In-silico

#### Docking results using AutoDock 1.5.7 software

**Figure 3:**
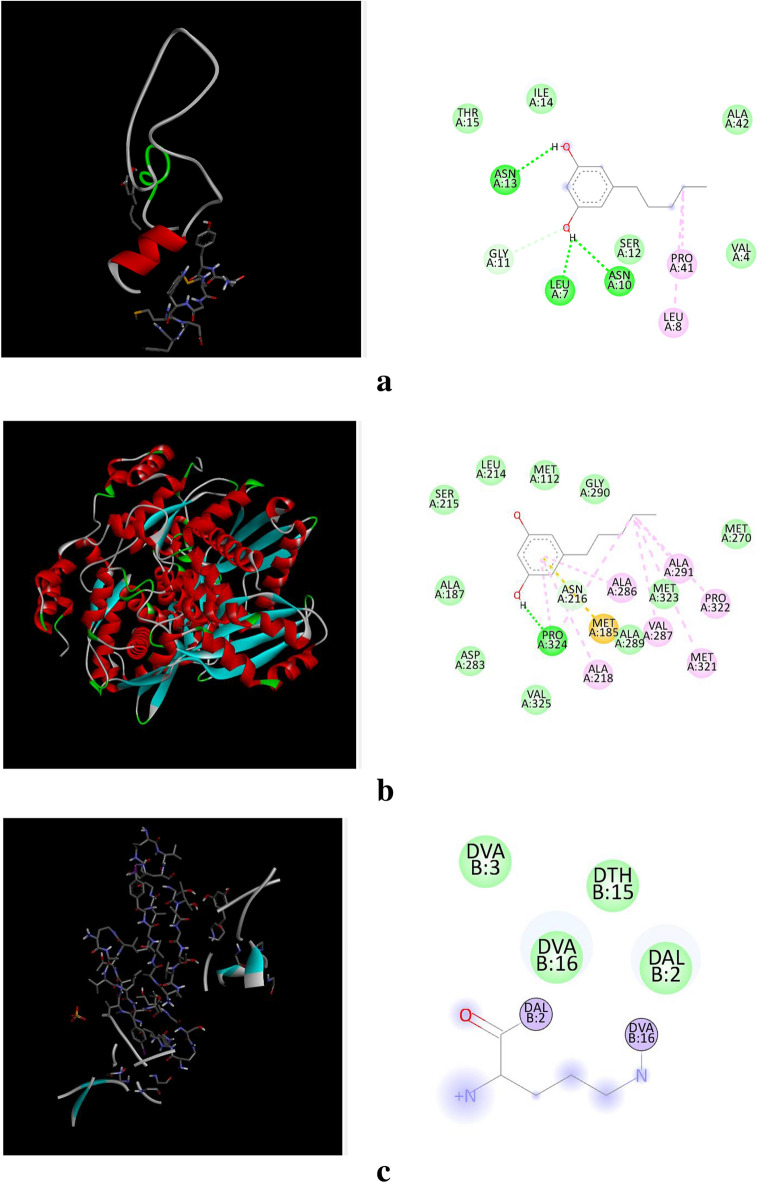

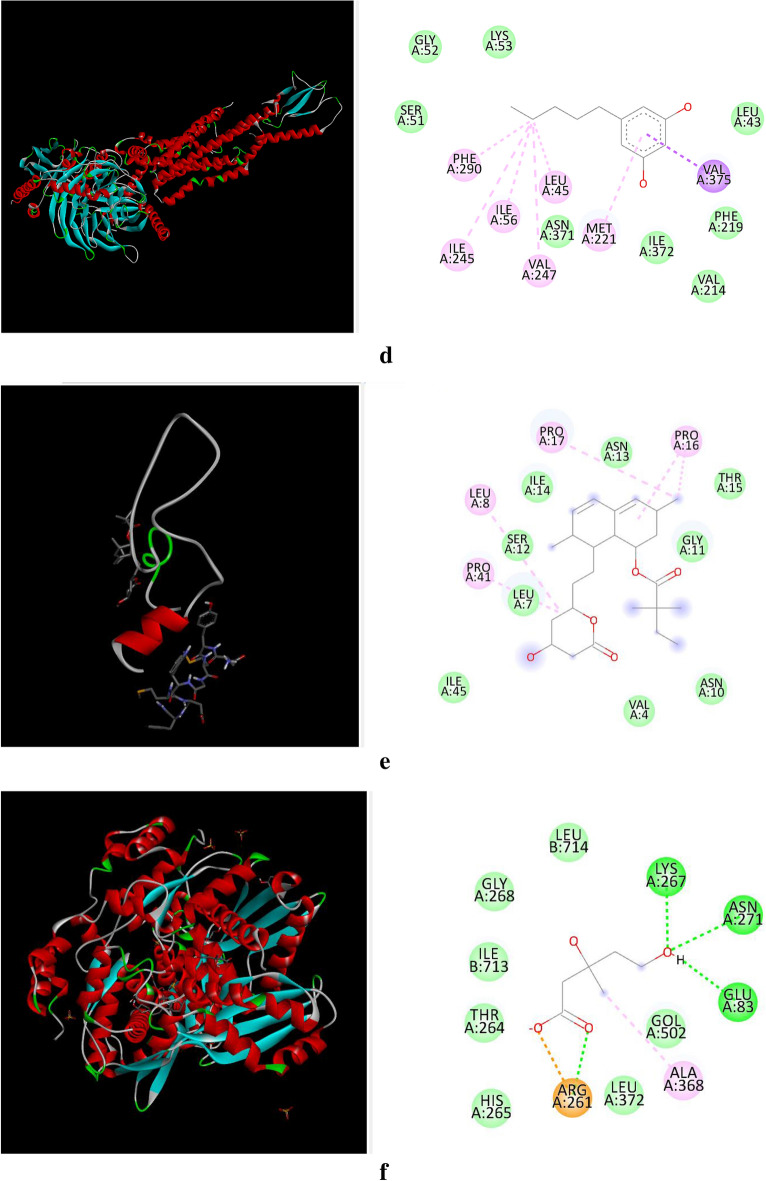

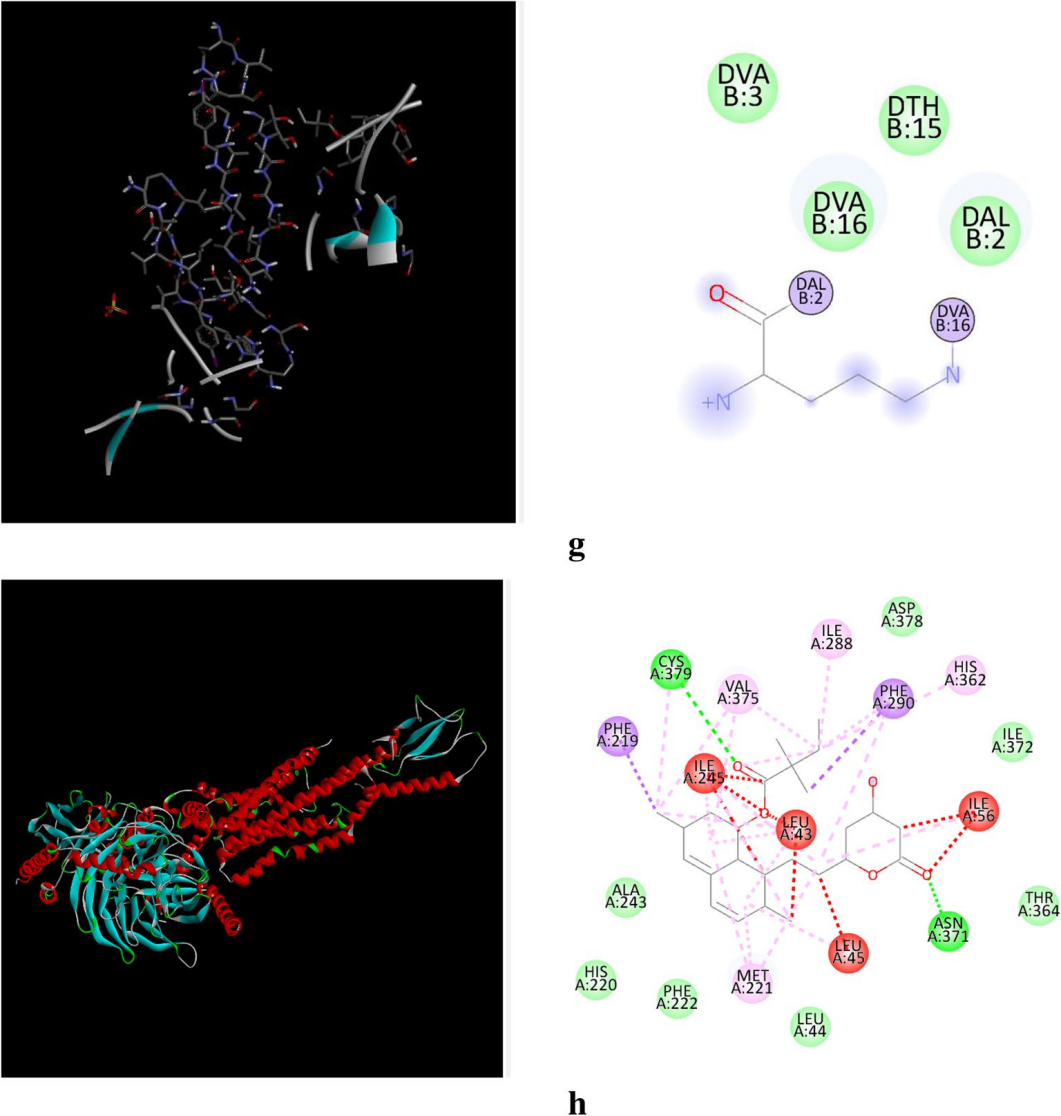
(**a**)–(**h**) Interaction of Olivetol & Simvastatin with CCK, HMG CoA, and GLP receptors. (**a**) Interaction of Olivetol with Cholecystokinin A Receptor. (**b**) Interaction of Olivetol with 3-hydroxy-3-methyl (HMG) Coenzyme A. (**c**) Interaction of Olivetol with Myeloperoxidase. (**d**) Interaction of Olivetol with Glucagon-Like Peptide-1. (**e**) Interaction of Simvastatin with Cholecystokinin A Receptor. (**f**) Interaction of Simvastatin with 3-hydroxy-3-methyl (HMG) Coenzyme A. (**g**) Interaction of Simvastatin with Myeloperoxidase. (**h**) Interaction of Simvastatin with Glucagon-Like Peptide-1.

**Figure 4:**
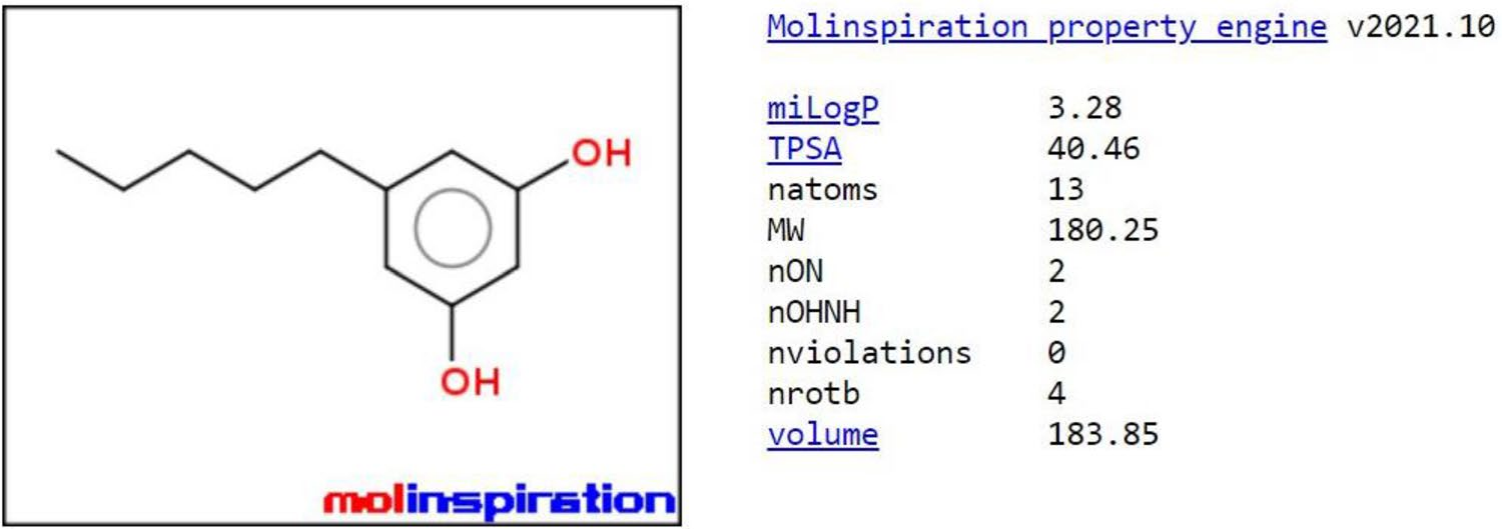
Molinspiration molecular properties of Olivetol.

The docking analysis was performed for Simvastatin and Olivetol against four different target proteins under the prescribed parameters. The interaction of Olivetol and Simvastatin with HMG-CoA is shown in Figs. 3 and 4. Olivetol was docked with predicted targets such as CCK, HMG CoA, and GLP receptors, which are involved in anti-obesity activity and are recorded in Table 2.

**Table 2:** Table of docking results using Autodock.

S.No	Proteins	PDB ID	Binding score for Simvastatin	Binding score for Olivetol
1	3-Hydroxy-3-methyl (HMG) Coenzyme A	4I6Y	− 7.42	− 5.05
2	Cholecystokinin A Receptor	1D6G	− 6.07	− 4.46
3	Glucagon-Like Peptide-1	5VAI	− 7.26	− 4.42
4	Myeloperoxidase	5FIW	− 6.03	− 4.41

### Pharmacokinetic analysis

#### Prediction of molecular properties and bioactivity score

**Figure 5:**
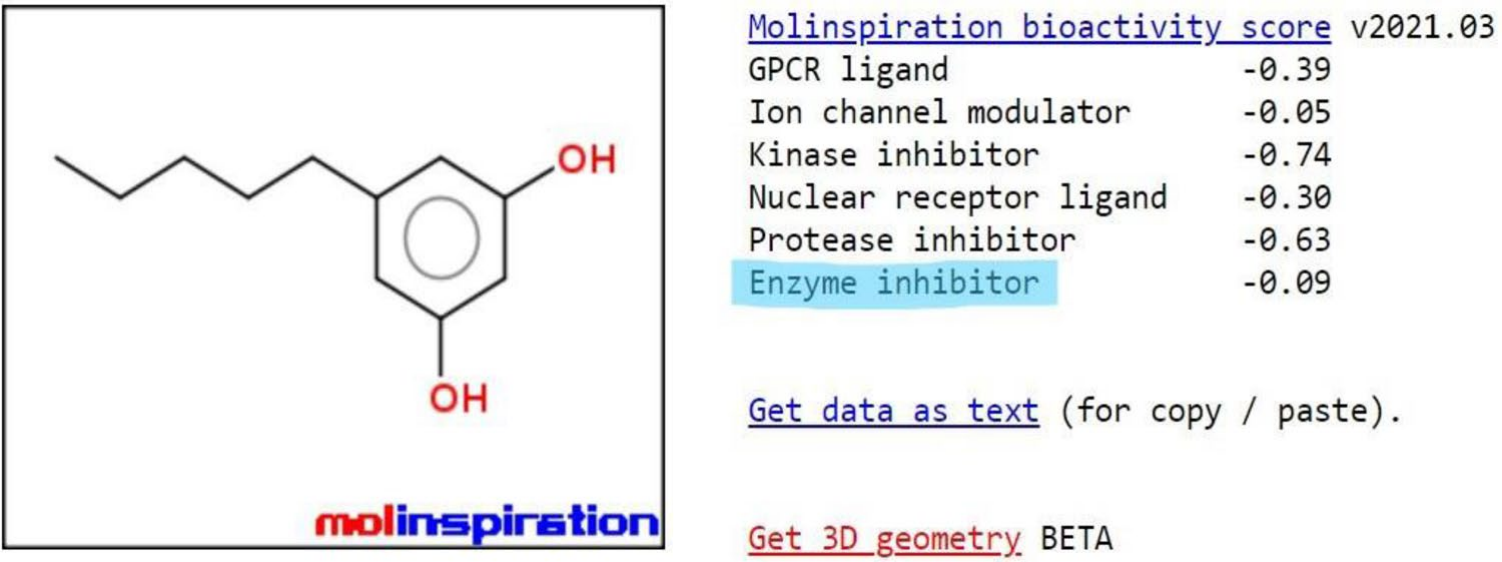
Bioactivity score of Olivetol.

**Figure 6:**
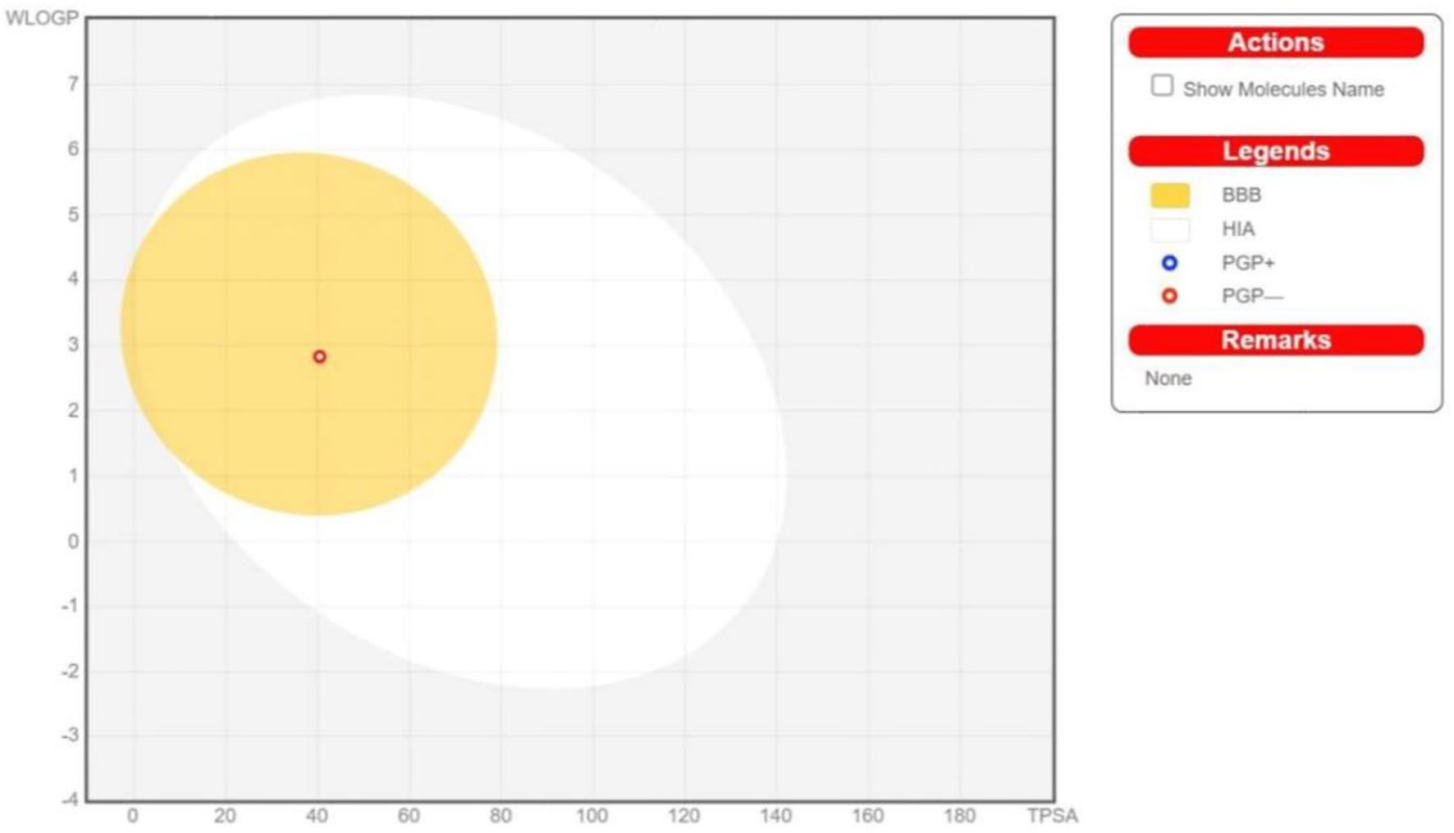
Graphical representation of BOILED-Egg which is an intuitive method to predict simultaneously two key ADME parameters, i.e. passive gastrointestinal absorption and brain access (BBB).

A graphical plot within the pinkish area predicts that Olivetol has excellent bioavailability, with significant absorption and distribution throughout the body. Based on the bioactivity score, Olivetol is predicted to treat obesity by acting as an enzyme inhibitor. The test drug also has effective blood–brain barrier penetration and gastrointestinal absorption capabilities. It is considered a drug-like compound as it satisfies Lipinski’s rule. The significant outcomes for Olivetol were obtained using in-silico tools such as Molinspiration cheminformatics, and the results from SwissADME are shown in Figs. 3, 4, 5, 6 and Tables 3, 4, 5, 6, 7, 8.

**Table 3:** Physicochemical properties of olivetol using the SwissADME web tool.

Physicochemical Properties	
Formula	C11H16O2
Molecular weight	180.24 g/mol
Num. heavy atoms	13
Num. arom. heavy atoms	6
Fraction Csp3	0.45
Num. rotatable bonds	4
Num. H-bond acceptors	2
Num. H-bond donors	2
Molar Refractivity	54.68
TPSA	40.46 A^2^

**Table 4:** Water solubility profile of Olivetol using the SwissADME web tool.

Water solubility	
Log S (ESOL)	− 3.2
Solubility	8.59e−02 mg/ml; 4.76e−04 mol/l
Class	Soluble
Log S (Ali)	− 4.17
Solubility	1.23e−02 mg/ml; 6.80e−05 mol/l
Class	Moderately soluble
Log S (SILOCOS-IT)	− 3.25
Solubility	1.01e−01 mg/ml; 560e−04 mol/l
Class	Soluble

**Table 5:** Lipophilicity of olivetol using the SwissADME web tool.

Lipophilicity	
Log P_o/w_ (iLOGP)	2.04
Log P_o/w_ (XLOGP3)	3.63
Log P_o/w_ (WLOGP)	2.83
Log P_o/w_ (MLOGP)	2.39
Log P_o/w_ (SILICOS-IT)	2.71
Consensus Log P_o/w_	2.72

**Table 6:** Pharmacokinetic properties of olivetol using the SwissADME web tool.

Pharmacokinetics	
GI Absorption	High
BBB permeant	Yes
P-gp substrate	No
CYP1A2 inhibitor	Yes
CYP2C19 inhibitor	No
CYP2C9 inhibitor	No
CYP2D6 inhibitor	Yes
CYP3A4 inhibitor	No
Log *k*_*p*_ (skin permeation)

**Table 7:** Druglikeness of olivetol using the SwissADME web tool.

Druglikeness	
Lipinski	Yes; 0 violation
Ghose	Yes
Verber	Yes
Egan	Yes
Muegge	No; 1 violation: MW < 200
Bioavailability score	0.55

**Table 8:** Medicinal chemistry properties of olivetol using the SwissADME web tool.

Medicinal chemistry	
PAINS	0 altert
Brenk	0 altert
Lead likeness	No; 2 violations: MW < 250, XLOGP3 > 3.5
Synthetic accessibility	1.47

#### Diet-induced obesity (DIO) model

**Figure 7:**
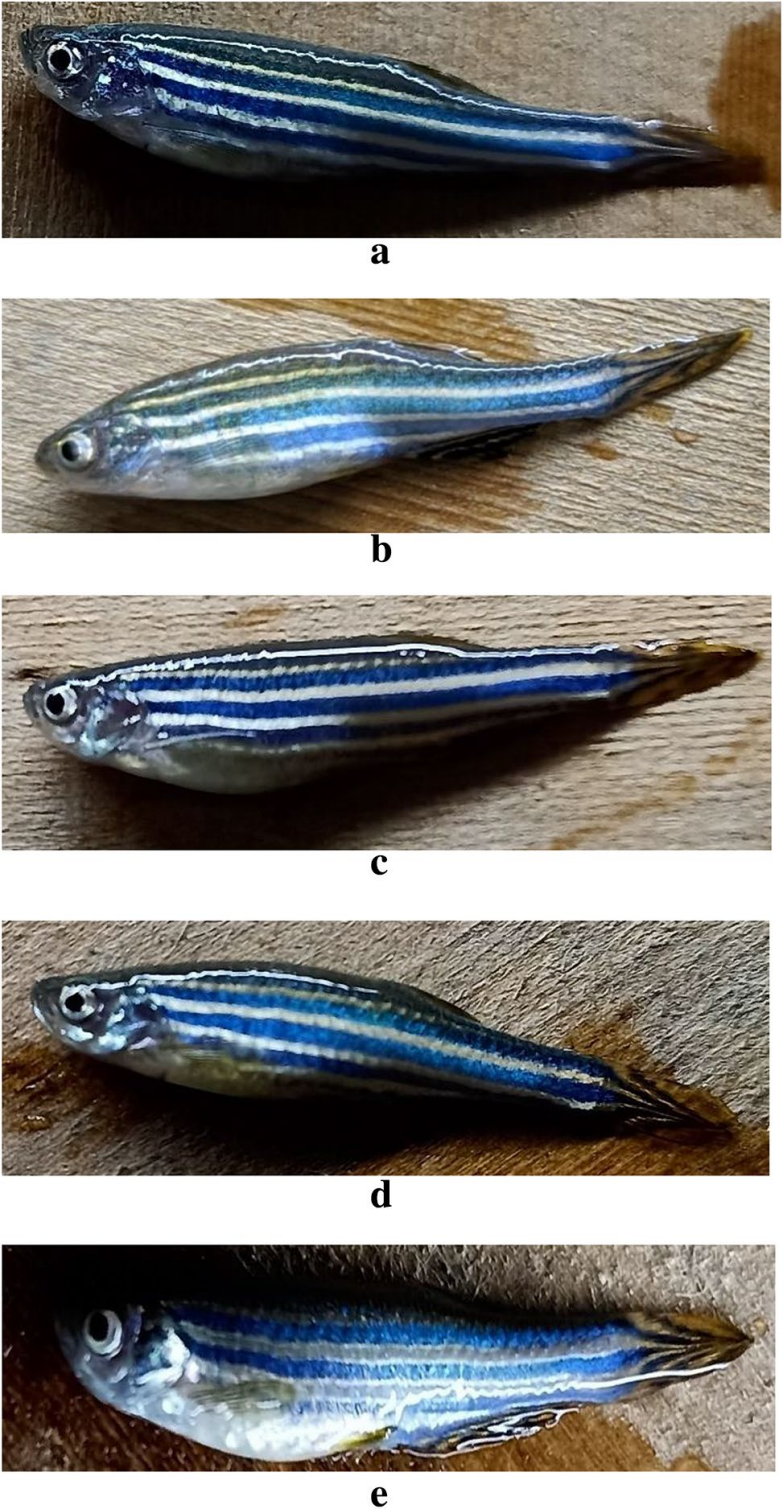
(**a**)–(**e**) Group of Adult Zebrafish. (**a**) Fish from group I, (**b**) Fish from group II, (**c**) Fish from group III, (**d**) Fish from group IV, (**e**) Fish from group V.

The zebrafish were made obese by feeding them a diet high in fat and calories. The body weight was assessed both before and following treatment with low and high concentrations of olive oil, and it was compared to both the groups receiving Simvastatin as the standard treatment and to the normal control. Figure 7b shows obesity when compared to the total body weights from other groups. Negative control groups exhibited significantly greater weight, which was alleviated by treatment with both low and high concentrations of Olivetol and by the standard drug Simvastatin.

#### Estimation of body weight

**Figure 8:**
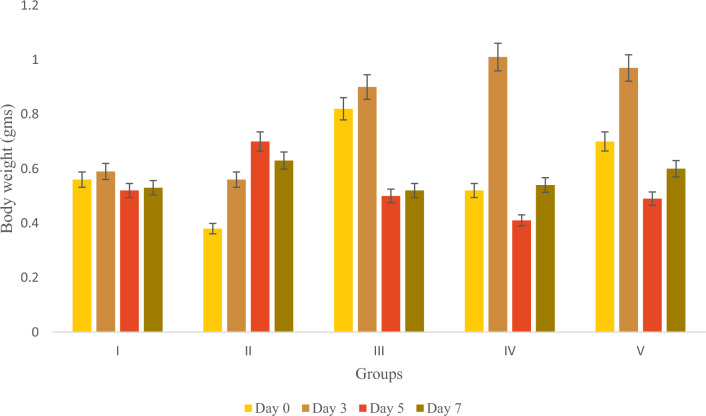
Average Body weight measurement of the groups. Data represented as Mean ± SD. *p* < 0.05 using one-way ANOVA.

The average body weight of zebrafish was measured and recorded in Table 9. The body weight of male and female zebrafish in the positive control group, classified as obese, was higher than that of the Olivetol-treated test groups. Figures 7 and 8 depict the body weight comparisons of different fish groups before and after treatment. The results indicate that the groups treated with Olivetol exhibited a dose-dependent decrease in body weight.

**Table 9:** Average measurement of body weight of the groups.

Group	Day 0 (gm)	Day 3 (gm)	Day 5 (gm)	Day 7 (gm)
I	0.56 ± 0.01	0.59 ± 0.02	0.52 ± 0.08	0.53 ± 0.01
II	0.38 ± 0.03	0.56 ± 0.03	0.70 ± 0.01	0.63 ± 0.03
III	0.82 ± 0.01	0.90 ± 0.04	0.50 ± 0.04	0.52 ± 0.05
IV	0.52 ± 0.02	1.01 ± 0.06	0.41 ± 0.03*	0.54 ± 0.07*
V	0.70 ± 0.01	0.97 ± 0.05	0.49 ± 0.01*	0.60 ± 0.05*

*Data represented as mean ± SD. *p* < 0.05 using one-way ANOVA.

#### Estimation of tissue cholesterol

**Figure 9:**
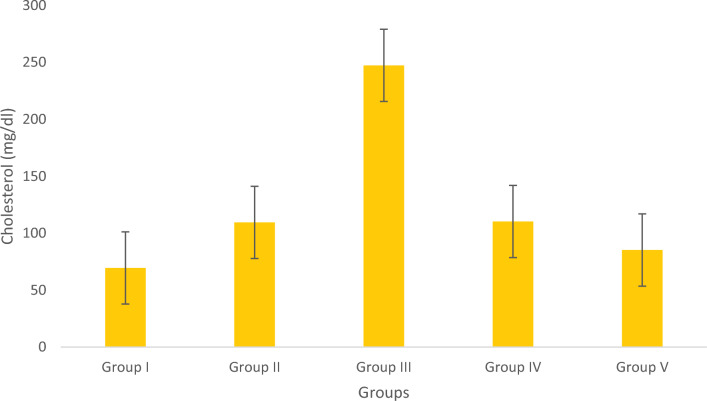
Estimation of Cholesterol. Data represented as Mean ± SD. *p* < 0.05 using one-way ANOVA.

Figure 9 provide a visual representation of the tissue cholesterol levels. The study compared the total cholesterol (TC) level between the standard and drug-treated groups. Results showed that the obese fish from the Olivetol-treated test groups displayed a dose-dependent decline in cholesterol levels, in contrast to the Simvastatin-treated negative control group.

#### Determination of triglycerides

**Figure 10:**
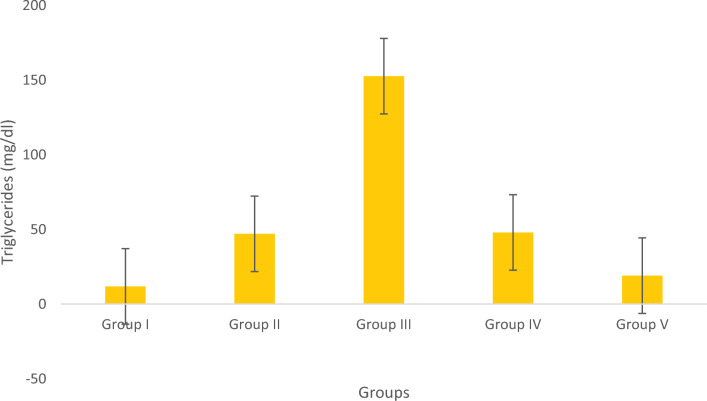
Estimation of Triglycerides. Data represented as Mean ± SD. *p* < 0.05 using one-way ANOVA.

Figure 10 display the visual representation of the TG levels. The results showed traces of higher TG levels in the obese group. However, fish treated with Olivetol exhibited decreased TG levels compared to the standard control group. Furthermore, following Olivetol administration, the TG levels significantly decreased in a dose-dependent manner.

#### Determination of HMG-CoA Reductase

**Figure 11:**
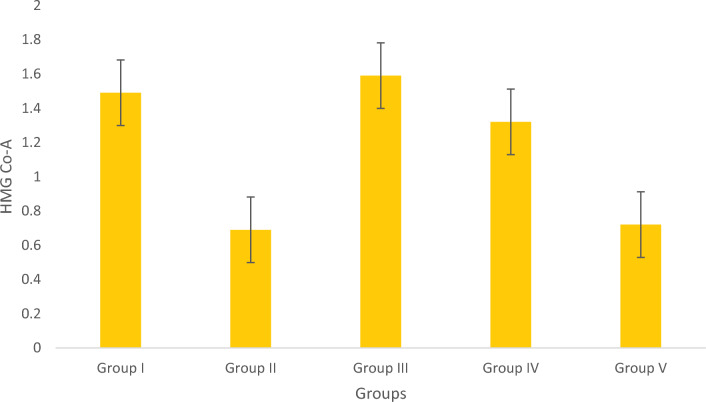
Estimation of HMG CoA. Data represented as Mean ± SD. *p* < 0.05 using one-way ANOVA.

The negative control group exhibited the least level of enzyme activity, but it was enhanced in the Simvastatin-treated group. Results depicted in Fig. 11 indicated that the positive control group was less effective in lowering the activity. In contrast, the groups treated with Olivetol caused a significant decrease in HMG-CoA reductase activity.

#### Assay of ALT

**Figure 12:**
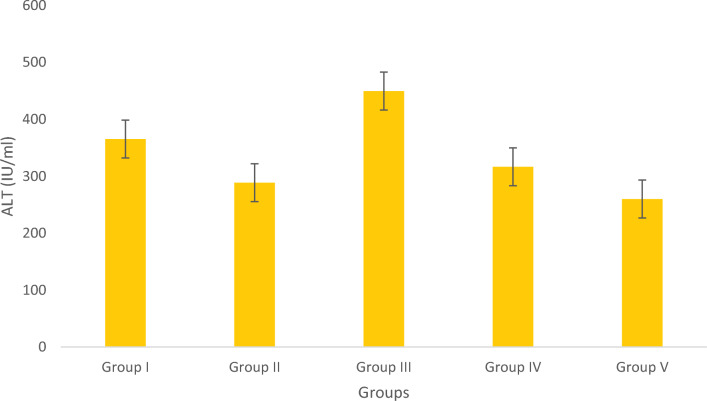
Estimation of ALT. Data represented as Mean ± SD. *p* < 0.05 using one-way ANOVA.

The levels of ALT were higher within the standard control group. The positive control group of obese fish had higher levels of ALT. The levels of ALT are graphically shown in Fig. 12. The obese groups were treated with conventional medicine, whereas the Olivetol-treated groups showed a dose-dependent reduction in the levels of ALT.

#### Assay of AST and ALP

**Figure 13:**
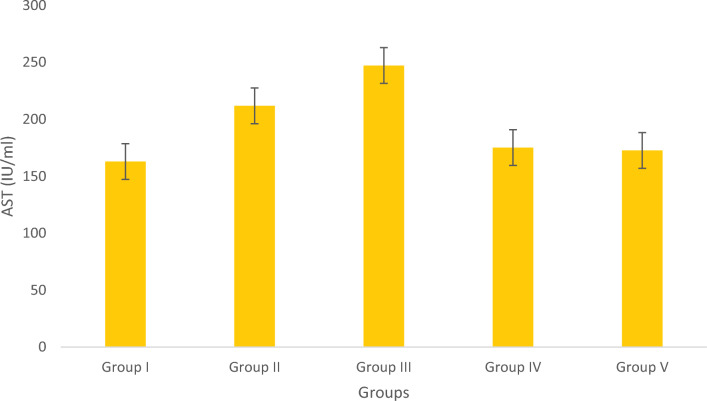
Estimation of AST. Data represented as Mean ± SD. *p* < 0.05 using one-way ANOVA.

**Figure 14:**
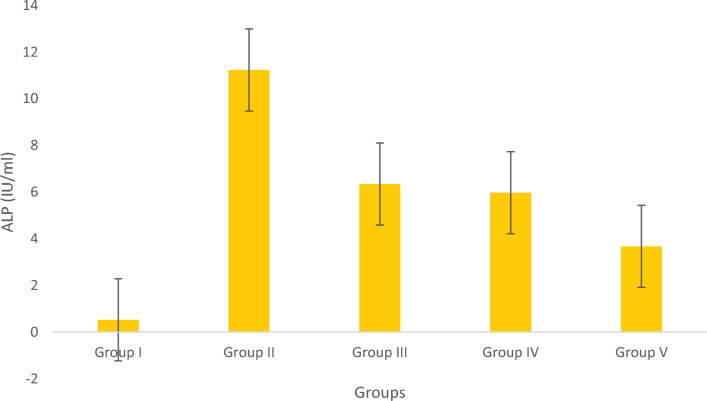
Estimation of ALP. Data represented as Mean ± SD. *p* < 0.05 using one-way ANOVA.

The AST level is shown in Fig. 13. The Obese fish from the positive control group had higher AST levels which were treated with Simvastatin. The levels for the groups receiving Olivetol-treatment decreased in a dose-dependent manner. The fishes treated with Olivetol had lower AST levels than fish in the control groups. The ALP levels were represented graphically in Fig. 14. The standard positive control group treated with Simvastatin showed higher ALP levels as compared to the Olivetol-treated test groups.

### Histopathology of zebrafish liver

**Figure 15:**
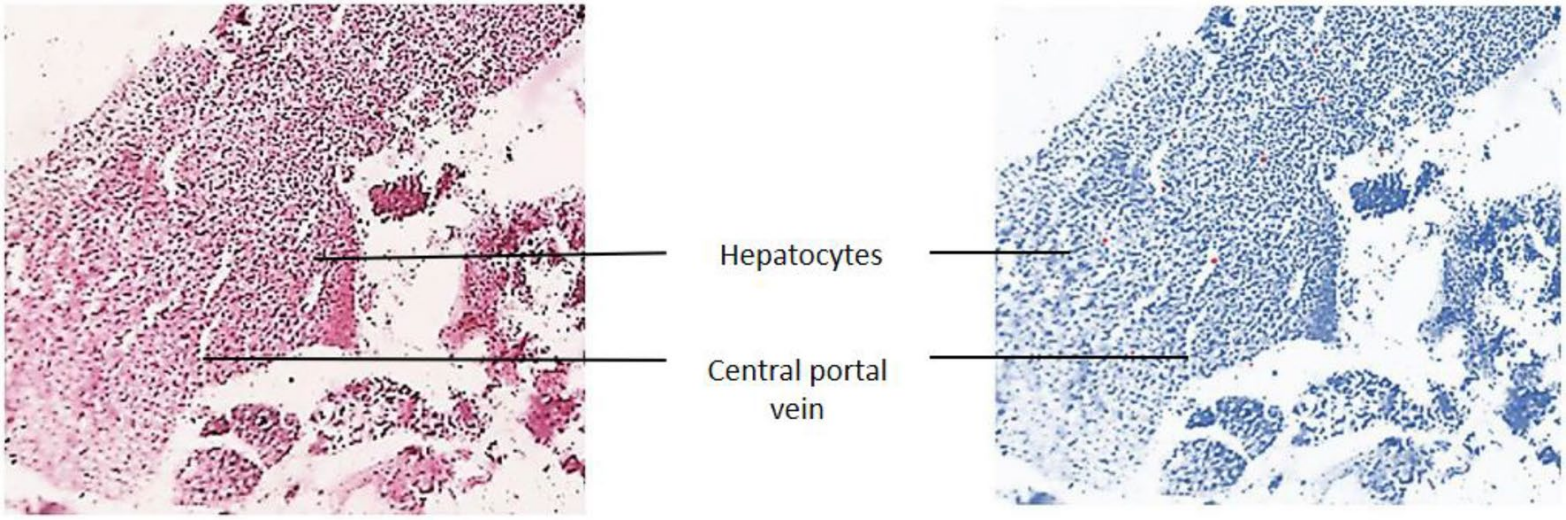
Liver section of the Group I (normal control).

**Figure 16:**
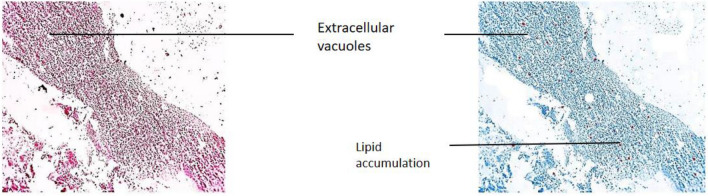
Liver section of Group II (negative control).

**Figure 17:**
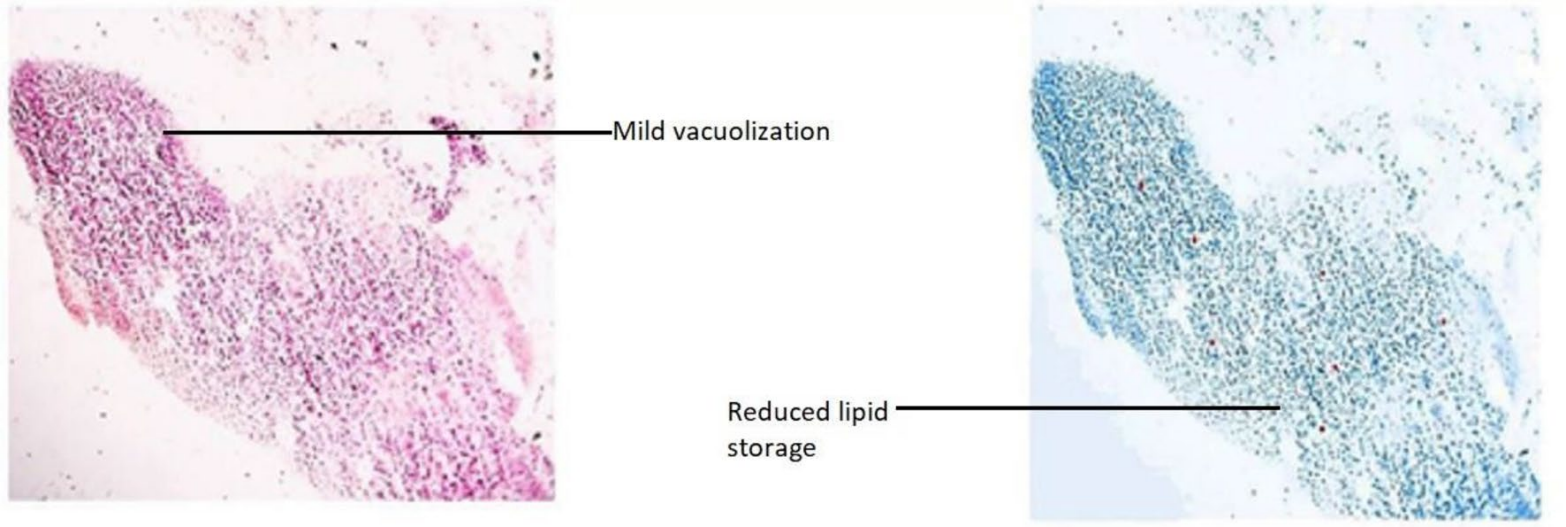
Liver section of Group III (positive control).

**Figure 18:**
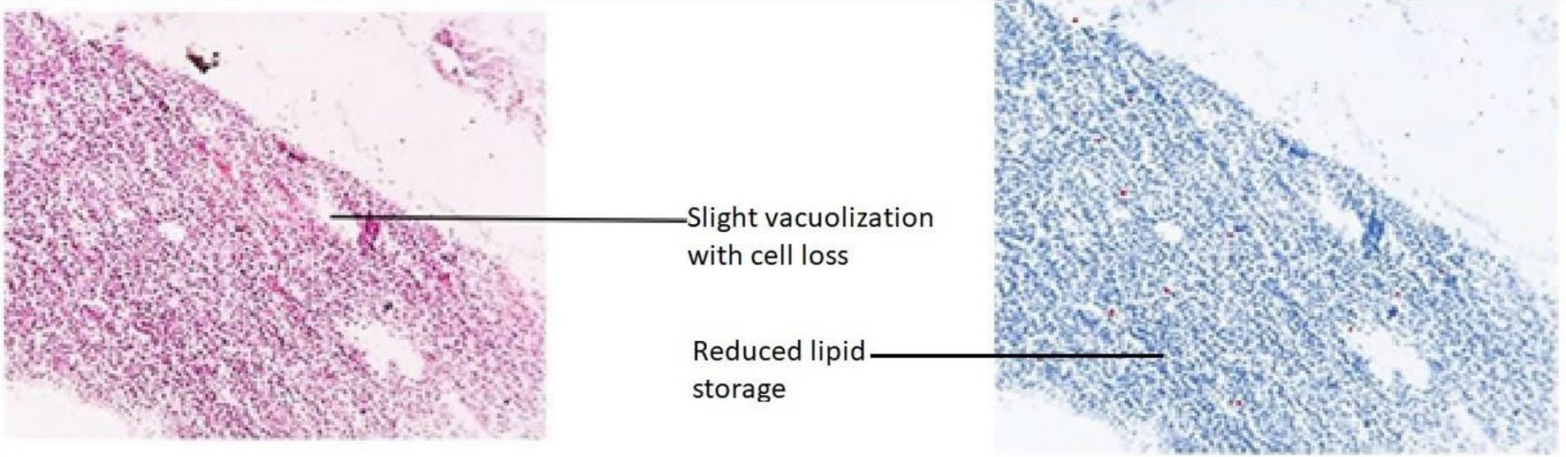
Liver section of Group IV (low dose of OL).

**Figure 19:**
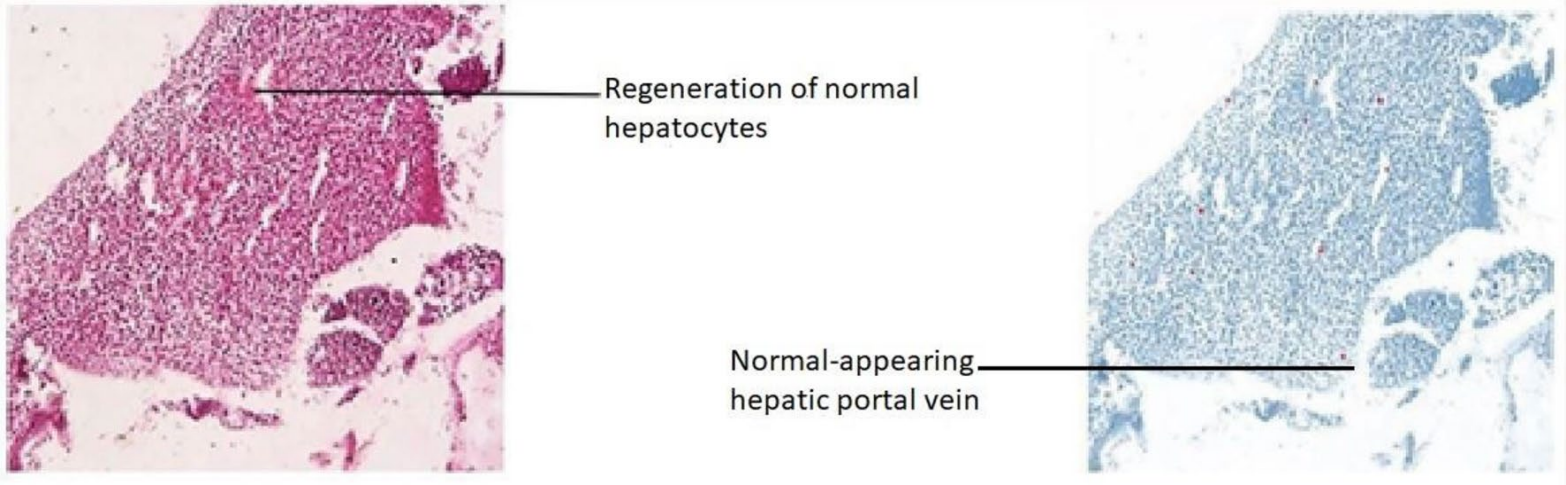
Liver section of High dose group V (high dose of OL).

The modifications detected in this organ also act as early indicators of harm to the animal’s health because they are typically simpler to spot than functional ones^53^. Hepatocytes emerging from the central portal vein were observed in Group-I. All the cells in the tissue were intact and plump (Fig. 15). In Group-II, contact between the cells became loose. Severe vacuolization was seen, with lipid accumulation more than 80% in extracellular vacuoles, and nucleus shrinkage (Fig. 16). Lipid accumulation was less (25%) compared to the negative and mild vacuolization was seen in Group-III (Fig. 17). In Group-IV, slight reduced vacuolization and cytoplasm loss were observed (Fig. 18). The histological score showed 55% lipid accumulation, with inflammation and vacuolization observed. In Group V, the normal hepatocytes regenerated and the hepatic portal vein appeared almost normal (Fig. 19). Compared to Group IV, the lipid content in the zebrafish liver of Group V was low with a lipid accumulation of 45%.

## Discussion

Obesity is a chronic condition that requires constant monitoring. It favors a chronic low-grade inflammatory state that is associated with metabolic dysfunction, multiple organ damage, and thrombotic disorders^54^. These physiological effects have a considerable impact on mortality and contribute significantly to the emergence of various morbidities, such as cardiovascular disease, type 2 diabetes, obstructive sleep apnea, and several malignancies^55^. Anti-obesity medications with higher effectiveness and better tolerability are required, especially when combined with dietary, behavioral, and exercise therapy. Sibutramine and orlistat have been shown to result in weight loss of up to 10%^56^. Recent advancements in the field of the peptidergic signaling of hunger and satiety from the gastrointestinal tract, which is mediated by ghrelin, HMG-CoA, CCK, and GLP-1, have unveiled new possibilities^57^. HMG-CoA reductase is the main enzyme in the cholesterol-producing mevalonate pathway, and its inhibition leads to reduced cholesterol generation in the liver. Statins, a class of synthetic medications, are frequently used to treat hypercholesterolemia. Phytoconstituents such as phenol 2,6-bis[1,1-dimethyl], 1-heptatriacotanol, oleic acid, eicosyl ester, naringin, apigenin, luteolin, ascorbic acid, and tocopherol have shown positive benefits in treating hypercholesterolemia and associated disorders^58^.

The current approach to address hypercholesterolemia primarily involves the administration of HMG-CoA reductase inhibitors. These medications function by obstructing cholesterol synthesis through the inhibition of HMG-CoA reductase in the liver, as well as facilitating the elimination of surplus cholesterol from the peripheral circulation through various mechanisms of reverse cholesterol transport. Cholecystokinin (CCK) is involved in promptly regulating appetite and energy consumption. Dysfunctions in CCK or its effects on gastrointestinal function and appetite could potentially contribute to the initiation and persistence of obesity. GLP-1, a peptide hormone consisting of either 30 or 31 amino acids, is secreted by the intestinal tract. This hormone enhances the release of insulin and lowers the concentration of glucagon under normal physiological circumstances. GLP-1 plays a critical role in maintaining normal glucose tolerance. GLP-1 actions are mediated by specific GLP-1 receptors (GLP-1Rs), which are part of the G-protein-coupled glucagon receptor family. These receptors are expressed in various tissues, including islet, small intestine, stomach, β-cells, mucosal cells, heart, and other cell types. Myeloperoxidase (MPO) is prominently present in neutrophils and has been associated with triggering the inflammatory response within adipose tissue. Hence, the study focused on investigating four receptors: HMG Co-A, CCK, GLP-1, and MPO.

The present study provides preliminary data that suggest Olivetol is capable of lowering cholesterol levels by inhibiting the activity of HMG-CoA reductase. In-silico molecular docking studies have been utilized to identify better binding scores for Olivetol with receptors such as CCK, GLP, and HMG Co-A. Based on the results of the in-silico molecular docking studies, Olivetol has shown better efficacy in these receptors. The pharmacokinetic study was performed using the preADMET web-based tool and the SwissADME web-based tool^59^. The bioavailability and molecular weight of Olivetol were estimated using a graphical RADAR plot. According to the molinspiration result, Olivetol has strong bioavailability, significant absorption, and dispersion in the body. Olivetol was primarily predicted to reduce obesity by inhibiting an enzyme, as indicated by the bioactivity score. It exhibits effective pharmacokinetic characteristics, including gastrointestinal absorption and blood–brain barrier re-penetration, and is considered to be drug-like as it satisfies Lipinski's rule. Olivetol showed positive results for drug-likeness, good bioavailability, absorption, and distribution, as well as a remarkable bioactivity score. In-vivo studies were performed in adult zebrafish with a diet-induced obesity (DIO) model to investigate the effects of Olivetol on the HMG-CoA receptor. Biomarkers associated with obesity were analyzed ex-vivo through biochemical and histopathological methods^60^.

The body weight of five groups of fish was measured before and after treatment with Olivetol, showing a significant reduction in weight.

Cholesterol, a fundamental constituent of all cells in the body, is synthesized within liver cells and acquired through dietary intake. Cholesterol commonly resides within the phospholipid bilayer of the cell membrane. Cholesterol serves as the precursor for all steroid hormones, cholesterol esters, and bile acids, while also functioning as a constituent of the plasma membrane. Due to its insoluble nature, cholesterol is transported throughout the body via lipoproteins. The lipoproteins encompass Low-Density Lipoprotein (LDL), High-Density Lipoprotein (HDL), and Very-Low Density Lipoproteins (VLDL). These lipoproteins package phospholipids, free cholesterol, and cholesterol esters, which are incorporated within VLDL. The quantification of total cholesterol within the bloodstream serves as an indicator of hyperlipidemia. Elevated cholesterol concentrations can restrict blood flow, consequently heightening the risk of coronary vascular diseases.

Reduced levels of HDL and elevated levels of triglycerides or LDL are also associated with dyslipidaemia, a distinctive feature in the progression of obesity. Persistent dyslipidemia plays a substantial role in the emergence of cardiovascular issues, notably atherosclerosis. In the ongoing investigation, a noteworthy elevation in total cholesterol and LDL levels has been identified, accompanied by an increase in HDL levels within the group of obese fishes.

In the present research, the administration of Olivetol treatment resulted in a reduction of total cholesterol levels, while simultaneously enhancing the levels of high-density lipoprotein (HDL) / good cholesterol. Considering that a decreased level of total HDL cholesterol is closely associated with the development of atherosclerosis, implementing therapeutic measures to elevate HDL-cholesterol can subsequently lead to a reduction in LDL-cholesterol. This beneficially impacts mitochondria function by aiding the conversion of food into energy. This approach also holds relevance in treating congenital heart disease, as it contributes to cholesterol metabolism.

Aminotransferase enzymes of clinical significance encompass ALT (Alanine Aminotransferase) and AST (Aspartate Aminotransferase). Levels of aspartate and alanine aminotransferase in the bloodstream can be utilized to assess instances of liver and heart injury^61^. Chronic kidney disease (CKD) has been linked to insulin resistance, which was associated with alanine aminotransferase (ALT) and aspartate aminotransferase (AST) to ALT ratio (AST/ALT ratio). Extensive research has been conducted regarding the levels of ALT and AST in blood serum. Building upon this, the current study aimed to investigate the concentrations of ALT in liver tissues and AST in kidney tissues.

Olivetol could be explored to understand its distinctive impact on enzymes in the liver and heart, as well as its influence on blood components. The hepatic enzymes, including Alanine aminotransferase (ALT), Aspartate aminotransferase (AST), and Gamma-glutamyl transferase (GGT), collectively fall under the category of transferases. This provides insight into the potential therapeutic application of Olivetol in case of liver-related conditions.

Previous research has indicated that animals exhibiting excessive body weight demonstrate various alterations in organs such as the heart, brain, liver, Kidneys as well as the testis in males and ovaries in females. This suggests that Olivetol has a significant effect on lipid profiles by reducing body weight and fat distribution in both male and female zebrafish. The estimation of total cholesterol (TC) in five groups of fish revealed that Olivetol has a significant effect in lowering TC levels. The assessment of triglyceride levels in five groups of fish showed a dramatic decrease after treatment with Olivetol. These results demonstrate that the test substance has a significant impact on reducing triglyceride levels.

The positive control group had significantly higher levels of HMG-CoA reductase compared to the Olivetol treated test groups. As a result, Olivetol was very effective in significantly decreasing the activity of the HMG CoA reductase. The positive control group of obese fish had higher levels of ALT. Olivetol-treated groups showed a dose dependent reduction.

The groups tested for AST levels resulted from the treatment with Olivetol having considerably lowered AST while having no effect on the standard group. This implies that Olivetol has a significant effect on AST. In comparison to the test groups, ALP the standard group was found to be significantly increased. For histological examination, zebrafish were euthanized using a higher concentration of MS-222, dissected, and tissues (liver) were fixed in fixative for further analysis. Dehydration of tissue was carried out using a graded series of ethanol, cleared in xylene, and embedded in paraffin. Sections of 1-4µm thickness were prepared from paraffin blocks using a microtome and then differential staining was performed using hematoxylin and eosin. To analyze lipid content in the tissues, oil red staining was performed. Histopathological changes were examined under a microscope. Histological alterations were scored as: (-) no histopathology; ( +) histopathology in < 25% of the field, (+ +) histopathology in > 75% of the field, and (+ + +) histopathology in all fields (Table 10).

**Table 10:** Histopathology scoring of oil red-stained liver tissue.

Group samples	Hisopathology scores
I (normal)	−
II (negative-Obesity)	+++
III (positive control of Simvastatin)	+
IV (low dose of OL)	++
V (high dose OL)	++

Hepatic cells with varying degrees of vacuolization in the cytoplasm were found in the livers of negative and test low zebrafish. The vacuolization on the H&E stained paraffin section was most likely caused by lipid droplet accumulation in the hepatocytes. In H&E stained sections of normal and standard zebrafish, hepatic lobules and cells showed well-preserved cytoplasm and a prominent nucleus. The liver of the zebrafish in test high had fewer vacuoles than the liver of the zebrafish in test low. However, mild hepatocellular inflammation caused by lipid accumulation was observed in both test low and high zebrafish liver tissue when compared to the standard. In normal liver, the cells were intact and plump. In the negative zebrafish liver, contact between cells became loose, the cytoplasm was lost, the nucleus shrank, and vacuoles formed. Massive red staining was observed in the negative liver, indicating the presence of lipid droplets. Lipid content was lower in the following order: standard, test high, and test low, when compared to negative. The zebrafish liver histopathology provided the observation of sinusoid spaces, hepatocytes, and vacuoles. High sinusoid spaces, moderate hepatocytes, and vacuoles were also observed in the high dose of olivetol. This proves that Olivetol has a significant impact on obesity and can be explored further for its anti-obesity potential using animal models.

## Conclusion

A dose-dependent decrease in triglycerides, HMG-CoA reductase, and cholesterol was observed after treatment with Olivetol. Olivetol was able to inhibit the storage of excess fat in the liver caused by obesity (steatohepatitis) by lowering the levels of liver enzymes. Based on the results of the biochemical tests and experimental data, it can be concluded that Olivetol has a beneficial impact on obesity. However, further research is required to determine the individual and/or synergistic effects of this compound.

## Data Availability

All data generated or analyzed during this study are included in this article.

## Abbreviations

ADME: Absorption, distribution, metabolism and excretion
ALP: Alkaline phosphatase
ALT: Alanine transaminase
AST: Aspartate transaminase
AT: Adipose tissue
BBB: Blood brain barrier
CCK: Cholecystokinin
CVD: Cardiovascular disease
DIO: Diet induced obesity
GI: Gastro-intestine
GLP: G-protein-coupled receptor
HDL-c: High density lipoprotein cholesterol
HFD: High fat diet
HMG-CoA: 3-Hydroxy-3 methyl glytaryl coenzyme A
LDL-c: Low density lipoprotein cholesterol
MPO: Myeloperoxidase
NFD: Normal fat diet
OSA: Obstructive sleep apnea
OL: Olivetol
PDB: Protein Data Bank
TC: Total cholesterol
TG: Triglyceride
T2D: Type 2 diabetes
